# Supplementary figures and images for: Multilevel geospatial analysis of factors associated with unskilled birth attendance in Ghana
Source: PLoS One. 2021 Jun 25;16(6):e0253603. doi: 10.1371/journal.pone.0253603 (PMC8232528; doi:10.1371/journal.pone.0253603)

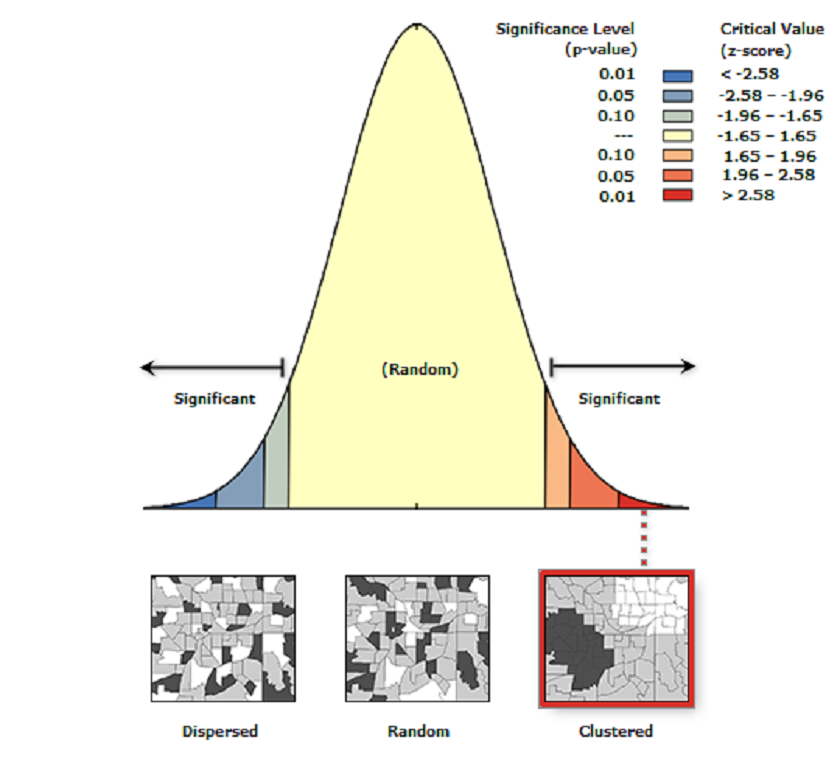


S1 Appendix: Spatial autocorrelation of unskilled birth in Ghana.

Source: GDHS, 2014

Supplement: S1 Appendix — Source: GDHS, 2014. (DOCX) [file pone.0253603.s001.docx]
