# Supplementary material for: Multilevel geospatial analysis of factors associated with unskilled birth attendance in Ghana
Source: PLoS One. 2021 Jun 25;16(6):e0253603. doi: 10.1371/journal.pone.0253603 (PMC8232528; doi:10.1371/journal.pone.0253603)
Supplement: S2 Appendix — Source: GDHS, 2014. (DOCX) [file pone.0253603.s002.docx]

S2 Appendix: OLS Diagnostics

| Number of observations | 190 | Akaike's Information Criterion (AICc) | -96.171034 |
| --- | --- | --- | --- |
| Multiple R-Squared | 0.352 | Adjusted R-Squared | 0.335 |
| Joint F-Statistic | 20.022 | Prob(>F), (5,184) degrees of freedom | 0.000000* |
| Joint Wald Statistic | 107.666 | Prob(>chi-squared), (5) degrees of freedom | 0.000* |
| Koenker (BP) Statistic | 23.211 | Prob(>chi-squared), (5) degrees of freedom | 0.000* |
| Jarque-Bera Statistic | 4.349 | Prob(>chi-squared), (2) degrees of freedom | 0.114 |

Source: GDHS, 2014.
