# Supplementary material for: Multilevel geospatial analysis of factors associated with unskilled birth attendance in Ghana
Source: PLoS One. 2021 Jun 25;16(6):e0253603. doi: 10.1371/journal.pone.0253603 (PMC8232528; doi:10.1371/journal.pone.0253603)
Supplement: S3 Appendix — Source: GDHS, 2014. (DOCX) [file pone.0253603.s003.docx]

S3 Appendix: GWR model for unskilled birth attendance in Ghana

| **Explanatory variables** | **Distance to facility, media exposure, NHIS subscription, low community economic status, and low community literacy** |
| --- | --- |
| Bandwidth | 134872.038 |
| Residual Squares | 3.618 |
| Effective Number | 44.647 |
| Sigma | 0.158 |
| AICc | -128.805 |
| R^2^ | 0.623 |
| R^2^ Adjusted | 0.509 |

Source: GDHS, 2014
