# Supplementary material for: Multilevel geospatial analysis of factors associated with unskilled birth attendance in Ghana
Source: PLoS One. 2021 Jun 25;16(6):e0253603. doi: 10.1371/journal.pone.0253603 (PMC8232528; doi:10.1371/journal.pone.0253603)
Supplement: Attachment — Submitted filename: PONE-D-21-07780_reviewer_Kelley.pdf [file pone.0253603.s005.pdf]

PLOS ONE

Multilevel Geospatial Analysis of unskilled birth attendant in Ghana

--Manuscript Draft--

**Manuscript Number:**	PONE-D-21-07780
**Article Type:**	Research Article
**Full Title:**	Multilevel Geospatial Analysis of unskilled birth attendant in Ghana
**Short Title:**	Multilevel Geospatial Analysis of unskilled birth attendant in Ghana
**Corresponding Author:**	Vincent Bio Bediako, MPhil<br>University of Cape Coast<br>Cape Coast, Central GHANA
**Keywords:**	Geospatial<br>Unskilled birth attendant<br>Public health<br>Social demography
**Abstract:**	Background: Globally, about 810 women die every day due to pregnancy and its related complications. Although the death of women during pregnancy or childbirth has declined from 342 deaths to 211 deaths per 100,000 live births between 2000 and 2017, maternal mortality is still higher particularly in sub-Saharan Africa and South Asia where 86% of all deaths occur.<br>Methods: A secondary analysis was carried out using the 2014 Ghana Demographic and Health Survey. A sample total of 4,290 women who had a live birth in the 5 years preceding the survey was included in the analysis. ArcGIS software was used to explore the spatial distribution of unskilled birth attendance in Ghana. The Geographic Weighted Regression (GWR) was employed in modeling spatial relationships. Moreover, a multilevel binary logistic regression model was fitted to identify factors associated with unskilled birth attendance.<br>Results: In this study, unskilled birth attendance had spatial variations across the country. The hotspot, cluster and outlier analysis identified the concerned districts in the north-eastern part of Ghana. The GWR analysis identified different predictors of unskilled birth attendance across districts of Ghana. In the multilevel analysis, mothers with no education, no health insurance coverage, and mothers from households with lower wealth status had higher odds of unskilled birth attendance. Being multi and grand multiparous, perception of distance from the health facility as not a big problem, urban residence, women residing in communities with medium and higher poverty level had lower odds of unskilled birth attendance.<br><br>Conclusion: unskilled birth attendance had spatial variations across the country. Areas with high levels of non-skilled birth attendance had mothers who had no formal education, not health insured, mothers from poor households and communities, Primiparous women, mothers from remote and border districts could get special attention in terms of allocation of resources including skilled human power, and improved access to health facilities
**Order of Authors:**	Vincent Bio Bediako, MPhil
	Ebenezer N. K. Boateng
	Bernard Afriyie Owusu
	Kwamena Sekyi Dickson
**Additional Information:**	
**Question**	**Response**
**Financial Disclosure**<br><br>Enter a financial disclosure statement that describes the sources of funding for the work included in this submission. Review	The authors received no specific funding for their work

Powered by Editorial Manager® and ProduXion Manager® from Aries Systems Corporation

the submission guidelines for detailed requirements. View published research articles from *PLOS ONE* for specific examples.<br><br>This statement is required for submission and **will appear in the published article** if the submission is accepted. Please make sure it is accurate.<br><br>**Unfunded studies**<br>Enter: *The author(s) received no specific funding for this work.*<br><br>**Funded studies**<br>Enter a statement with the following details:<br>• Initials of the authors who received each award<br>• Grant numbers awarded to each author<br>• The full name of each funder<br>• URL of each funder website<br>• Did the sponsors or funders play any role in the study design, data collection and analysis, decision to publish, or preparation of the manuscript?<br>• **NO** - Include this sentence at the end of your statement: *The funders had no role in study design, data collection and analysis, decision to publish, or preparation of the manuscript.*<br>• **YES** - Specify the role(s) played.<br><br>* typeset	
**Competing Interests**<br><br>Use the instructions below to enter a competing interest statement for this submission. On behalf of all authors, disclose any competing interests that could be perceived to bias this work—acknowledging all financial support and any other relevant financial or non-financial competing interests.<br><br>This statement is required for submission and **will appear in the published article** if the submission is accepted. Please make sure it is accurate and that any funding sources listed in your Funding Information later in the submission form are also	The authors have declared that no competing interest exist

declared in your Financial Disclosure statement.<br><br>View published research articles from *PLOS ONE* for specific examples.<br><br>**NO authors have competing interests**<br><br>Enter: *The authors have declared that no competing interests exist.*<br><br>**Authors with competing interests**<br><br>Enter competing interest details beginning with this statement:<br><br>*I have read the journal's policy and the authors of this manuscript have the following competing interests: [insert competing interests here]*<br><br>* typeset	
**Ethics Statement**<br><br>Enter an ethics statement for this submission. This statement is required if the study involved:<br><br>• Human participants<br>• Human specimens or tissue<br>• Vertebrate animals or cephalopods<br>• Vertebrate embryos or tissues<br>• Field research<br><br>Write "N/A" if the submission does not require an ethics statement.<br><br>General guidance is provided below. Consult the submission guidelines for detailed instructions. **Make sure that all information entered here is included in the Methods section of the manuscript.**	N/A

**Format for specific study types**<br><br>**Human Subject Research (involving human participants and/or tissue)**<br>• Give the name of the institutional review board or ethics committee that approved the study<br>• Include the approval number and/or a statement indicating approval of this research<br>• Indicate the form of consent obtained (written/oral) or the reason that consent was not obtained (e.g. the data were analyzed anonymously)<br><br>**Animal Research (involving vertebrate animals, embryos or tissues)**<br>• Provide the name of the Institutional Animal Care and Use Committee (IACUC) or other relevant ethics board that reviewed the study protocol, and indicate whether they approved this research or granted a formal waiver of ethical approval<br>• Include an approval number if one was obtained<br>• If the study involved *non-human primates*, add *additional details* about animal welfare and steps taken to ameliorate suffering<br>• If anesthesia, euthanasia, or any kind of animal sacrifice is part of the study, include briefly which substances and/or methods were applied<br><br>**Field Research**<br><br>Include the following details if this study involves the collection of plant, animal, or other materials from a natural setting:<br>• Field permit number<br>• Name of the institution or relevant body that granted permission	
**Data Availability**<br><br>Authors are required to make all data underlying the findings described fully available, without restriction, and from the time of publication. PLOS allows rare exceptions to address legal and ethical concerns. See the PLOS Data Policy and FAQ for detailed information.	Yes - all data are fully available without restriction

A Data Availability Statement describing where the data can be found is required at submission. Your answers to this question constitute the Data Availability Statement and **will be published in the article**, if accepted.<br><br>**Important:** Stating 'data available on request from the author' is not sufficient. If your data are only available upon request, select 'No' for the first question and explain your exceptional situation in the text box.<br><br>Do the authors confirm that all data underlying the findings described in their manuscript are fully available without restriction?	
**Describe where the data may be found in full sentences. If you are copying our sample text, replace any instances of XXX with the appropriate details.**<br><br>• If the data are **held or will be held in a public repository**, include URLs, accession numbers or DOIs. If this information will only be available after acceptance, indicate this by ticking the box below. For example: *All XXX files are available from the XXX database (accession number(s) XXX, XXX.).*<br>• If the data are all contained **within the manuscript and/or Supporting Information files**, enter the following: *All relevant data are within the manuscript and its Supporting Information files.*<br>• If neither of these applies but you are able to provide **details of access elsewhere**, with or without limitations, please do so. For example:<br><br>*Data cannot be shared publicly because of [XXX]. Data are available from the XXX Institutional Data Access / Ethics Committee (contact via XXX) for researchers who meet the criteria for access to confidential data.*<br><br>*The data underlying the results presented in the study are available from (include the name of the third party*	All data used in this manuscript will be found on http://www.measuredhs.com/

and contact information or URL).<br>• This text is appropriate if the data are owned by a third party and authors do not have permission to share the data.<br><br>* typeset	
Additional data availability information:	Tick here if the URLs/accession numbers/DOIs will be available only after acceptance of the manuscript for publication so that we can ensure their inclusion before publication.; Tick here if your circumstances are not covered by the questions above and you need the journal's help to make your data available.

Powered by Editorial Manager® and ProduXion Manager® from Aries Systems Corporation

Click here to access/download;Manuscript;Final_Work_Unskilled_Birth.docx

Multilevel Geospatial Analysis of unskilled birth attendant in Ghana

Vincent Bio Bediako[1*], Ebenezer N. K. Boateng[2], Bernard Afriyie Owusu[1], Kwamena Sekyi Dickson[1]

[1]Department of Population and Health, University of Cape Coast, Ghana
[2]Department of Geography and Regional Planning, University of Cape Coast

***Corresponding author**

Email addresses:

VBB: vincent.bediako@stu.ucc.edu.gh
ENKB: ebenezer.boateng@stu.ucc.edu.gh (https://orcid.org/0000-0002-3911-5345)
BAO: bernard.owusu@stu.ucc.edu.gh
KSD: nadicx@gmail.com (https://orcid.org/0000-0002-3152-2317)

Abstract

Background: Globally, about 810 women die every day due to pregnancy and its related complications. Although the death of women during pregnancy or childbirth has declined from 342 deaths to 211 deaths per 100,000 live births between 2000 and 2017, maternal mortality is still higher particularly in sub-Saharan Africa and South Asia where 86% of all deaths occur.

Methods: A secondary analysis was carried out using the 2014 Ghana Demographic and Health Survey. A sample total of 4,290 women who had a live birth in the 5 years preceding the survey was included in the analysis. Arc-GIS software was used to explore the spatial distribution of unskilled birth attendance in Ghana. The Geographic Weighted Regression (GWR) was employed in modeling spatial relationships. Moreover, a multilevel binary logistic regression model was fitted to identify factors associated with unskilled birth attendance.

Results: In this study, unskilled birth attendance had spatial variations across the country. The hotspot, cluster and outlier analysis identified the concerned districts in the north-eastern part of Ghana. The GWR analysis identified different predictors of unskilled birth attendance across districts of Ghana. In the multilevel analysis, mothers with no education, no health insurance coverage, and mothers from households with lower wealth status had higher odds of unskilled birth attendance. Being multi and grand multiparous, perception of distance from the health facility as not a big problem, urban residence, women residing in communities with medium and higher poverty level had lower odds of unskilled birth attendance.

Conclusion: Unskilled birth attendance had spatial variations across the country. Areas with high levels of non-skilled birth attendance had mothers who had no formal education, not health insured, mothers from poor households and communities, Primiparous women, mothers from remote and border districts could get special attention in terms of allocation of resources including skilled human power, and improved access to health facilities.

Keywords: Geospatial, unskilled birth attendant, public health, social demography

Background

Globally, about 810 women die every day due to pregnancy and its related complications (41). Although the death of women during pregnancy or childbirth has declined from 342 deaths to 211 deaths per 100,000 live births between 2000 and 2017, maternal mortality is still higher particularly in sub-Saharan Africa and South Asia where 86% of all deaths occur. As such, the lifetime risk of obstetric causes of maternal deaths in developing countries is 33 times higher than deaths in developed countries. Evidence depicts that 94 percent of maternal and child deaths occur in low-and-middle-income countries including Ghana (41). However, Ghana has chalked major successes in its quest to reduce maternal and neonatal mortality. For instance, the maternal mortality ratio (MMR) has declined from 760 deaths per 100,000 live births in 1990 to 350/100,000 livebirths in 2010, and 319/100,000 livebirths in 2015 (36). Similarly, neonatal deaths (NMR) have steadily declined from 59 deaths per 1,000 live births in 1970 to 23 per 1,000 live births in 2019. These reductions are also due to the substantial increase in healthcare institutional deliveries from 46% in 2003 to 78% in 2018 (36). However, births assisted by skilled personnel in Ghana are below 70%, compared to the global 81 percent coverage (13).

Skilled birth attendance (SBA) is defined as births assisted by healthcare trained professionals such as doctors, midwives, and nurses to manage uncomplicated pregnancies, childbirth, the postpartum period (within 24 hours), and new-born health (40). SBA has been identified as one of the surest ways of extenuating the risks of maternal deaths during pregnancy (13). It is therefore recommended that SBA be relied upon throughout the continuum of care as it has clinical rationales – prevention of direct and indirect obstetric complications including haemorrhage, eclampsia, obstetric fistula, obstructed labour, unsafe abortions (28; 16), and HIV and malaria, respectively. Further, neonatal deaths are lowest in countries with the highest coverage of skilled birth attendance (25), and it is found that SBA reduces stillbirth by 23% (30).

In Ghana, appreciable progress has been made to promote SBA. The National Health Insurance Scheme (NHIS)for instance covers SBA, and pregnant women are further exempt from paying NHIS premiums (8). Considerable improvements have also been achieved in Ghana's Universal access to healthcare as Community-based Health Planning Services (CHPS) have been extended to reach hitherto remote areas in the country. It is therefore not surprising that SBA has increased from 44% in 1993 to 68% in 2011 (24). However, MMR of 319/100,000 and NMR of 30 deaths per 1,000 live births are still unexpectedly high, and a major contributor of MMR and NMR is unskilled birth attendance, defined as births assisted by a traditional birth attendant (TBA), traditional health volunteer, community/village health volunteer, neighbours/ friends/relatives, and other untrained individuals (5). Such women are usually assisted by either traditional birth attendants (TBAs) or relatives and friends (2), and this has dire consequences for both the mother and her unborn child or children. Unskilled birth attendance is undoubtedly a major drawback to achieving SDG 3 target 3.1, which aims at reducing the global maternal mortality ratio to less than 70 per 100,000 (15).

Several factors have been identified in Ghana to influence women's decision to utilize unskilled birth attendance. These factors include low socio-economic status, no national health insurance (NHIS) coverage, residing in rural areas, higher-order parity, ethnicity, traditional religion, previous contact with the health system, and low levels of education (14; 5; 21). Budu (14) for instance found that as parity increased, home delivery was more likely to occur, and women with no NHIS were more likely to deliver at home. In other reports, Apanga and Awoonor-Williams (9) found that physical inaccessibility to health care, socio-cultural and economic

factors, and health care system challenges were the major causes of low levels of skilled birth attendance in Ghana. In his equity analysis also, Zere et al. (45) showed that many maternal health services including access to SBA and caesarean section services, healthcare facilities, use of modern contraceptives, and intermittent treatment for malaria are not equitably distributed as women in wealthier households are more likely to utilize MCH services, compared to their counterparts from poorer households. Evidence of North-South variations in SBA has been reported as well.

Skilled birth attendance (SBA) uptake is higher in southern Ghana (14; 1; 4), compared to Northern parts of Ghana (17). Such variations and uneven use of services blurs national progress and continuously perpetuates inequalities in health. Cues must be taken from the fact that Ghana failed to achieve some MDG targets including those related to maternal health. We, therefore, analyse the current Demographic and Health Survey data of Ghana to determine individual, community, and regional levels factors predicting unskilled birth attendance in Ghana. We further contribute to the maternal and child health literature in Ghana by highlighting hotspots for unskilled birth attendance and the factors associated with such observations. Previous geospatial studies have either focused on inadequate variables (10) or focused on skilled birth attendance (19). Understanding the predictors of unskilled birth attendance and its geospatial coverage in Ghana is of high relevance to both local and public health understanding of unskilled birth attendance, as well as highlight areas where national interventions will yield cost-effective results.

Methods

Source of data

The Ghana Demographic and Health Survey (GDHS) used a standard DHS model questionnaire developed by the Measure DHS programme. This study used the most recent DHS data and a cross-sectional study design. The DHS are national surveys carried out every five years in low-and middle-income countries globally. The surveys concentrate on maternal and child health, physical activity, sexually transmitted infections, fertility, health insurance, tobacco use, and alcohol consumption. They provide data to monitor the demographic and health profiles of the respective countries. For the study, women with birth history who had given birth up to five years before the survey were included. A sample of 4,290 women with complete data required for our analysis participated in this study. Permission to use the data set was given by the MEASURE DHS following the assessment of our concept note. The DHS program permitted us to use the dataset after the evaluation of our concept note. The datasets are freely available to the public at www.measuredhs.com.

Description of variables

Outcome variables

Outcome variable

The main outcome variable was unskilled birth attendance. The outcome variable was derived from the response to the question "who assisted with the delivery?" Responses were categorized under Health Personnel and Other Person. Health personnel included doctor, nurse, nurse/midwife, auxiliary midwife, and other people which consisted of a traditional birth attendant (TBA), traditional health volunteer, community/village health volunteer, neighbours/

friends/relatives, others. For this study, unskilled birth attendance referred to births assisted by a traditional birth attendant (TBA), traditional health volunteer, community/village health volunteer, neighbours/ friends/relatives, other (6).

Explanatory variables

Eleven explanatory variables were used. These were grouped into individual and community level variables. Individual characteristics (level of education, age, parity, health insurance coverage, wealth status, distance to a health facility, and media exposure), community-level characteristics (Type of residence, Community socioeconomic status, Community literacy, Region of residence)

Statistical Analysis

We employed both descriptive and inferential analytical approaches. First, we computed the proportion of women who utilized the service of unskilled birth attendants during delivery. This ensued with bivariate analysis between individual characteristics (level of education, age, parity, health insurance coverage, wealth status, distance to a health facility, and media exposure), community characteristics (Type of residence, Community socioeconomic status, Community literacy, Region of residence) and utilization of unskilled birth attendants (see Table 1). Following the hierarchical nature of the data set, the Multilevel Logistic Regression Model (MLRM) was employed. This comprises fixed effects, and random effects (11). The fixed effects of the model were gauged with binary logistic regression which resulted in odds ratios (ORs) and adjusted odds ratios (aORs) (see Table 2). The random effects on the other hand were assessed with Intra-Cluster Correlation (ICC) (23) (see Table 2). The sample weight (v005/1,000,000) was applied in all the analyses to control for over and under-sampling. All the analyses were carried out using STATA version 14.

Model fit and specifications.

We assessed the fitness of all the models with the Likelihood Ratio (LR) test. The presence of multicollinearity between the independent variables was checked before fitting the models. The variance inflation factor (VIF) test revealed the absence of high multicollinearity between the variables (Mean VIF= 2.28).

Spatial Analysis

In the conduct of the survey, instead of mapping-out houses in which the data was collected, clusters were mapped to protect the actual identity and location of respondents (32). These clusters are developed to suit the district-level data. Thus, making it easy to merge the household records with spatial data. During the period of the data collection, there were 216 administrative districts in Ghana however not all districts had respondents drawn from for the survey. This aided in the merger of the data gathered with the district shapefiles obtained from the Department of Geography and Regional Planning, University of Cape Coast, Ghana. This was done to permit the analysis to be made at a district level. The data is best analysed at the district level since the information is more representative at the cluster level (32).

Therefore, this study extracted the required variables from the 2014 GDHS. The extracted data maintained the mapped clusters information. This mapped cluster information was used to help

join the extracted non-spatial data to the coordinates gathered for the clusters. To aid in the spatial analysis, all the data required (GDHS data and 216 district boundary) were projected into the projected coordinate system of Ghana Meter Grid. The extracted GDHS data was merged with coordinate and afterward, a spatial join was undertaken to transfer the cluster point to the 216-district boundary (polygon) layer using ArcMap version 10.5. This activity enabled us to easily identify and trace where each case is located within a district. It was identified that some of the district boundaries had more than one cluster. In such cases, the data from the clusters were aggregated and their means were computed to represent the respective district they fell within (32).

With regards to the geospatial analyses, four spatial statistical tools were applied to analyse the data. These tools were spatial autocorrelation (Global Moran's I), hot spot analysis (Getis-Ord G), outlier and cluster analysis, and Geographically Weighted Regression. The spatial autocorrelation tool calculates the distribution (clustering or dispersion) of a phenomenon based on its geographical locations using a specific variable which is mostly the outcome variable (29; 32). The output of the spatial autocorrelation results is a z-score between -1 to +1 describing the degree of spatial concentration or dispersion for a measured variable (32). The spatial autocorrelation was, therefore, used to assess whether unskilled birth attendance in Ghana had a clustering or dispersion pattern at the district-level. This study hypothesized that unskilled birth attendance is randomly distributed across various districts in the country. The null hypothesis is rejected if a calculated p-value is small (95% confidence interval); which implies an unlikely situation that the observed spatial pattern is a result of random processes. Hence, a district with a high z-score and a small p-value indicates a spatial clustering of high values. The Moran's I identify clusters of features with values similar in magnitude. Moran's I values range from -1 (disperse) to +1 (clustered). A calculated Moran's I value of 0 indicates complete spatial randomness. This study used $p < 0.05$ to determine the statistical significance of the computed index values.

Further, hot spot analysis (Getis-Ord G) was used to ascertain statistically significant spatial variations in unskilled birth attendance (29; 32). The hot spot analysis tool uses continuous data such as the incidence of unskilled birth attendance to show hot and cold spots. This analysis was conducted to determine districts with high prevalence against areas of the low prevalence of unskilled birth attendance. The hot spot analysis compares the local mean rate to the global mean rate. Thus, this analysis tool compares the rate for a cluster with its nearest neighbour to the rest of all clusters. After the analysis, the output consists of a z-score and p-value for each cluster (district) which permits the assessment of the significance of differences between local and global means (29). A cluster (district) with a high positive z-score and a small p-value indicates a hot spot whereas a cluster (district) with a low negative z-score and a small p-value indicates a cold spot. A z-score near zero indicates no apparent spatial clustering. The output shows the statistical significance using confidence intervals of 99%, 95%, and 90%. The confidence level indicates the significance of the prevalence of short birth at the district level. An outlier and cluster analysis was conducted to identify districts that appeared as outliers. Outlier districts could either be a hot spot district that is surrounded by cold spot districts and vice-versa.

After ascertaining the hot spot analysis of unskilled birth attendance, the geographically weighted regression (GWR) modelling was conducted. This is a spatial regression modelling performed to identify which explanatory variables best accounts for the observed spatial

patterns of unskilled birth attendance. Before running the GWR, an ordinary least squares (OLS) regression was conducted to check the reliability of the explanatory variables if they satisfy all the assumptions that are required. The OLS in ArcGIS 10.5 was used to check the statistical significance of the explanatory variables. The explanatory variables with statistically significant coefficients were considered appropriate for the GWR. Finally, the geographically weighted regression (GWR) was analysed. The intuition around the GWR is to be able to use spatial modelling to explain the strength of a statistically significant explanatory variable. Thus, a variable that is a strong predictor in one cluster may not necessarily be a strong predictor in another cluster (29). To be specific, the GWR uses the OLS coefficient from the clusters in relation to its nearest neighbours in modelling the predictability of the explanatory variable. The output shows how the strength of each explanatory variable across space. Therefore, maps of the statistically significant coefficients were generated.

Results

Background characteristics and unskilled birth attendant

Forty – four percent of women with no education utilized the service of an unskilled birth attendant during delivery. A high proportion of women with grand multiparous (37.3%), poorest wealth status (50.4%), low media exposure (49.5%), and those who saw the distance to a health facility as a big problem (37.4%) utilized the service of an unskilled birth attendant during delivery (see Table 1). Three in ten women from rural areas and communities with low socioeconomic status utilized the service of an unskilled attendant during delivery (see Table 1).

Table 1: Background characteristics and unskilled birth attendant.

Variable	Frequency N=4,290	Percentage (%)	Proportion of unskilled birth attendant
**Individual Characteristics**			
Level of education			
No education	1,117	26.0	44.7
Primary	822	19.7	29.9
Secondary	2,133	49.7	13.0
Higher	198	4.6	1.3
Age			
15 – 19	190	4.5	21.1
20 – 24	728	17.0	25.5
25 – 29	1,037	24.2	22.0
30 – 34	1,006	23.4	25.1
35 – 39	808	18.8	20.5
40 – 44	396	9.2	27.8
45 – 49	125	2.9	39.3
Parity			
Primiparous	977	22.8	13.2
Multiparous	2,231	52.0	22.4
Grand multiparous	1,982	25.2	37.3
Covered by health insurance			

No	1,425	33.2	31.3
Yes	2,865	66.8	20.4
Wealth status			
Poorest	901	21.0	50.4
Poorer	871	20.3	37.3
Middle	857	20.0	21.7
Richer	844	19.7	5.1
Richest	817	19.0	
Getting medical help for self: distance to health facility			
Big problem	1,138	26.5	37.4
Not a big problem	3,152	73.5	19.2
Media exposure			
Low	405		49.5
Medium	1,107		33.2
High	2,778		16.7
**Community variables**			
Type of residence			
Urban	1,981	46.2	8.7
Rural	2,309	53.8	37.2
Community socioeconomic status			
Low	2,953	68.8	35.6
High	1,337	31.2	7.3
Community literacy			
Low	1,440	33.6	44.8
Medium	1,437	33.5	26.3
High	1,413	32.9	8.3
Region of residence			
Western	442	10.3	22.3
Central	472	11.0	27.3
Greater Accra	698	16.3	6.8
Volta	327	7.6	29.1
Eastern	403	9.4	30.7
Ashanti	764	17.8	12.4
Brong Ahafo	388	9.0	19.7
Northern	497	11.6	60.5
Upper east	184	4.3	15.2
Upper west	115	2.7	32.5

Multilevel Logistic regression of unskilled birth attendants

Results for all the models were presented in Table 2. From the final model (Model IV), our study found out that level of education, age, parity, health insurance coverage, wealth status, media exposure, place of residence, community literacy had a significant relationship with unskilled birth attendants.

Women with higher education had a lesser likelihood (aOR=0.07, CI=0.01, 0.55) of utilizing the services of unskilled birth attendants compared to those with no education. Primiparous women have a lesser odd (aOR=0.51, CI=0.38, 0.70) utilization of the service of unskilled birth attendants during delivery compared to multiparous women (see Table 2). Women age 35 – 39 years were less likely (aOR=0.64, CI=0.47, 0.87) to utilize the service of unskilled birth attendants compared to those 25 – 29 years. Women who were not covered by health insurance had a higher likelihood of utilizing delivery services from unskilled birth attendants (aOR=1.80, CI=1.48, 2.19) compared to those who were covered by health insurance. We also found that women with the richest wealth status had a lesser odd of utilizing delivery services from unskilled birth attendants (aOR=0.17, CI=0.08, 0.36) compared to those with the poorest wealth status (see Table 2). Women with low media exposure had a higher likelihood of utilizing delivery services from unskilled birth attendants (aOR=1.35, CI=1.01, 1.80) compared to those with high media exposure (see Table, 2).

Furthermore, women from urban centres had a lesser odd of utilizing delivery services from unskilled birth attendants (aOR=0.52, CI=0.35, 0.77) compared to those from the rural areas. Women from communities with low literacy had a higher odd of utilizing delivery services from unskilled birth attendants (aOR=2.06, CI=1.32, 3.23) compared to those from communities with high literacy level (see Table 2).

Table 2: Multilevel Logistic regression of unskilled birth attendants among women in Ghana

**Variables**	**Model 1**	**Model II aOR (95% CI)**	**Model III aOR (95% CI)**	**Model IV aOR (95% CI)**
Level of education				
No education		Ref		Ref
Primary		0.77**(0.60, 0.97)		0.81(0.63, 1.03)
Secondary		0.49***(0.38, 0.63)		0.52***(0.40, 0.67)
Higher		0.07*(0.01, 0.53)		0.07*(0.01, 0.55)
Age				
15 – 19		1.01(0.62, 1.66)		1.01(0.62, 1.66)
20 – 24		0.89(0.67, 1.19)		0.90(0.67, 1.20)
25 – 29		Ref		Ref
30 – 34		0.82(0.63, 1.08)		0.87(0.66, 1.14)
35 – 39		0.60**(0.44, 0.82)		0.64**(0.47, 0.87)
40 – 44		0.59**(0.40, 0.85)		0.65**(0.45, 0.94)
45 – 49		0.71(0.43, 1.16)		0.75(0.46, 1.23)
Parity				
Primiparous		0.49***(0.36, 0.66)		0.51***(0.38, 0.70)
Multiparous		Ref		Ref
Grand multiparous		1.30*(1.01, 1.67)		1.26(0.97, 1.62)

Covered by health insurance				
No		1.81***(1.49, 2.19)		1.80***(1.48, 2.19)
Yes		Ref		Ref
Wealth status				
Poorest		Ref		Ref
Poorer		0.80(0.63, 1.03)		0.78(0.60, 1.01)
Middle		0.46***(0.34, 0.63)		0.53***(0.38, 0.70)
Richer		0.10***(0.06, 0.16)		0.16***(0.10, 0.27)
Richest		0.08***(0.04, 0.16)		0.17***(0.08, 0.36)
Getting medical help for self: distance to health facility				
Big problem		1.21(0.99, 1.48)		1.10(0.90, 1.35)
Not a big problem		Ref		Ref
Media exposure				
Low		1.42*(1.06, 1.89)		1.35*(1.01, 1.80)
Moderate		0.99(0.80, 1.24)		0.96(0.77, 1.20)
High		Ref		Ref
Community level				
Place of Residence				
Urban			0.33***(0.23, 0.48)	0.52**(0.35, 0.77)
Rural			Ref	Ref
Community socioeconomic status				
Low			Ref	Ref
High			0.48**(0.30, 0.77)	0.83(0.50, 1.34)
Community literacy				
Low			3.91***(2.51, 6.10)	2.06**(1.32, 3.23)
Medium			2.25***(1.54, 3.28)	1.60*(1.10, 2.34)
High			Ref	Ref
Region of residence				
Western			0.99(0.49, 2.04)	0.92(0.45, 1.89)
Central			1.44(0.72, 2.89)	1.07(0.53, 2.17)
Greater Accra			Ref	Ref
Volta			1.75(0.85, 3.60)	1.17(0.56, 2.37)
Eastern			1.64(0.81, 3.32)	1.16(0.56, 2.37)
Ashanti			0.72(0.35, 1.48)	0.59(0.28, 1.23)
Brong Ahafo			0.62(0.30, 1.29)	0.42*(0.21, 0.91)
Northern			3.13**(1.15, 6.47)	1.58(0.75, 3.36)
Upper east			0.40*(0.19, 0.85)	0.20***(0.09, 0.43)
Upper west			0.79(0.37, 1.69)	0.49(0.22, 1.07)

**Random effect result**				
PSU variance (95% CI)	2.65(2.09, 3.35)	1.17(0.89, 1.54)	0.85(0.62, 1.15)	0.71(0.51, 0.98)
ICC	0.45	0.26	0.20	0.18
LR Test	χ^2=854.06 p=0.0000	χ^2=281.75 p=0.0000	χ^2=185.05 p=0.0000	χ^2=130.24 p=0.0000
Wald chi-square		356.45	302.07	463.11
Model fitness				
Log-likelihood	-2082.78	-1864.82	-1940.67	-1810.84
BIC	4182.28	3905.29	4006.80	3906.06
AIC	4169.55	3771.65	3911.33	3690
N	4,290	4,290	4,290	4,290

*p<0.05 **p<0.01 *** p<0.001

Spatial distribution on unskilled birth attendants

The spatial distribution of unskilled birth attendance results from Moran's I spatial autocorrelation analysis (Figure 1) revealed that unattended skilled birth was clustered. This implies that unskilled birth attendance was not randomized. This, therefore, led to exploring further to visualize the distribution of unskilled birth attendance in Ghana using Hotspot Analysis (GetisOrd Gi).

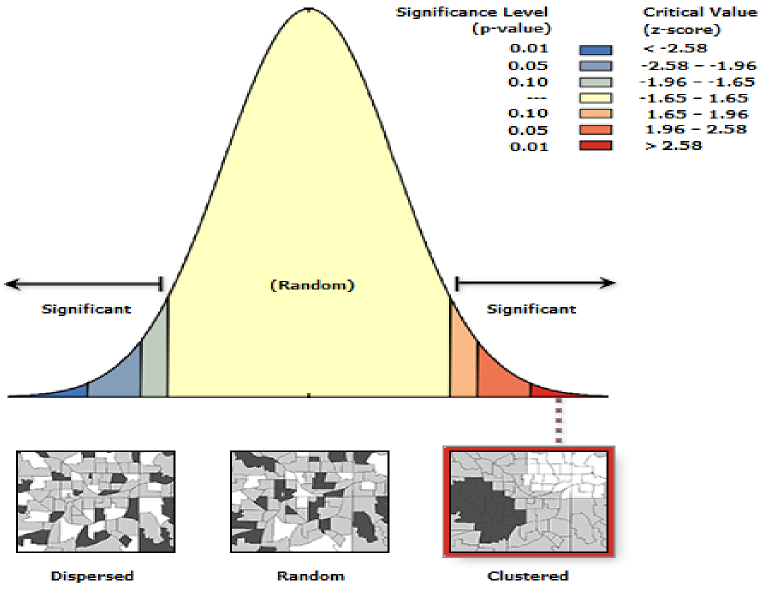

Fig 1: Spatial autocorrelation of unskilled birth in Ghana.

Source: Produced from GDHS, 2014

The hotspot analysis shows areas of statistically significant high and low intensity of the distribution of a phenomenon under study. According to Fig 2, there is a statistically significant (99% confidence level) high clustering of unskilled birth attendance in some parts of north-eastern Ghana (areas shown in red colour). However, some parts of the south showed a cold spot indicating high skilled birth attendance (areas shown in blue colour). Although the hotspot analysis identified statistically significant clustered areas, the cluster and outlier analysis were conducted to show districts that may have high unskilled birth attendance but were not captured in the hotspot analysis. The hotspot analysis does a cluster analysis and zones areas as hot or cold spots when an area has a similar occurrence of a phenomenon with its neighbours. This necessitated the conduct of the cluster and outlier analysis to identify outlier areas.

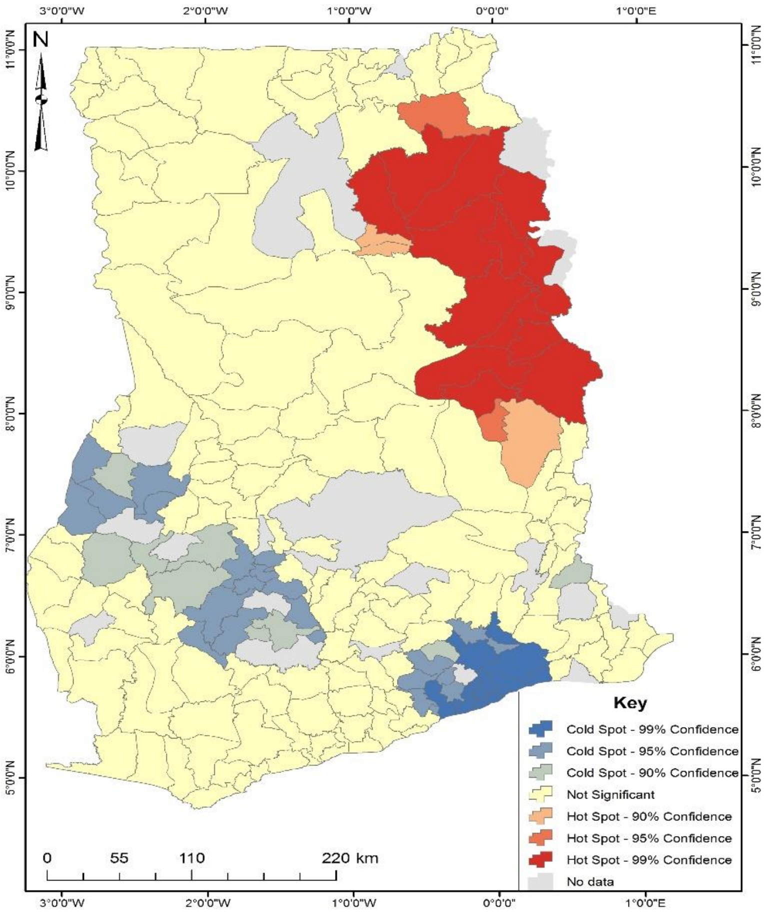

Fig 2: Hotspot analysis of unskilled birth attendance in Ghana

Source: Produced from GDHS, 2014

The cluster and outlier analysis (Figure 3) revealed that about 13 districts (marked in the colour red) that are randomly distributed have high unskilled birth attendance but could not be captured as a hotspot since there were found closer to areas with low unskilled birth attendance. Also, 2 districts (marked in colour blue) were found to have low unskilled birth attendance but closer to districts with high unskilled birth attendance. This result reveals that relying on hotspot analysis is good considering areas with statistically significant similar characteristics

to their neighbours whereas cluster and outlier analysis aids in identifying areas statistically significant distinctive characteristics.

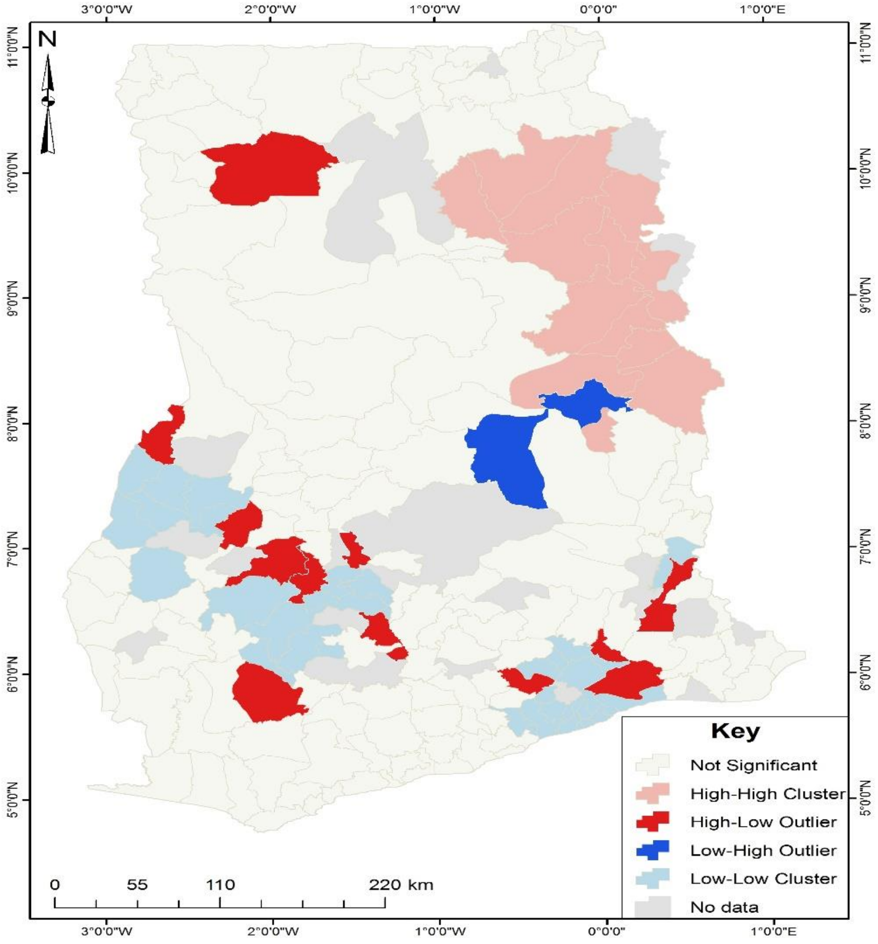

Fig 3: Cluster and outlier analysis of unskilled birth attendance in Ghana.

Source: Produced from GDHS, 2014

Further analysis was conducted to assess factors that account for the spatial distribution of unskilled attendance in Ghana. These factors were assessed using the Geographically Weighted Regression (GWR) analysis. However, to conduct GWR, the ordinary least square (OLS) analysis was conducted to identify significant factors that can explain the existing distribution of unskilled birth attendance in Ghana. The robust probability results of the OLS (Table 3) revealed that four out of five factors could geographically explain the spatial distribution of unskilled birth attendance in Ghana.

Table 3: OLS of factors that accounts for unskilled birth attendance in Ghana.

**Variable**	**Coefficient**	**StdError**	**t-Statistic**	**Probability**	**Robust_SE**	**Robust_T**	**Robust Probability**
Intercept	-0.013	0.038	-0.335	0.738	0.031	-0.409	0.683
Distance to Facility	0.272	0.057	4.789	0.000*	0.062	4.403	0.000*

Media exposure	0.214	0.101	2.118	0.036*	0.121	1.767	0.079
NHIS Subscription	0.193	0.068	2.837	0.0053*	0.068	2.86	0.005*
Community economic status	0.122	0.043	2.857	0.005*	0.032	3.761	0.000*
Community literacy	0.097	0.037	2.619	0.010*	0.046	2.115	0.036*

Source: Computed from GDHS, 2014.

Table 4: OLS Diagnostics

Number of observations	190	Akaike's Information Criterion (AICc)	-96.171034
Multiple R-Squared	0.352	Adjusted R-Squared	0.335
Joint F-Statistic	20.022	Prob(>F), (5,184) degrees of freedom	0.000000*
Joint Wald Statistic	107.666	Prob(>chi-squared), (5) degrees of freedom	0.000*
Koenker (BP) Statistic	23.211	Prob(>chi-squared), (5) degrees of freedom	0.000*
Jarque-Bera Statistic	4.349	Prob(>chi-squared), (2) degrees of freedom	0.114

Source: Computed from GDHS, 2014.

The four significant predictors identified from the OLS results were used to conduct the Geographically Weighted Regression (GWR) analysis (see Table 4). The GWR is a local model that estimates the different responses of the dependent variable on independent variables based on locational effects. Table 3 shows the GWR model for unskilled birth attendance in Ghana. Since GWR localizes the results of a phenomenon under study, it improves the model fit when the relationship between the predictors and short birth interval is non-stationary (25). This is evident from the R^2 and adjusted R^2 values where the OLS value were 0.352 and 0.335 whilst the GWR values were 0.623 and 0.509, respectively. The GWR's adjust R^2 value explains about 50% of the phenomenon under study (see Table 5). The subsequent fig 4-7 show the spatial predictive power of the explanatory variables towards the unskilled birth attendance in Ghana. Areas shaded red have a strong predictive power while areas shaded blue have a low predictive power of the explanatory variable.

Table 5: GWR model for unskilled birth attendance in Ghana

**Explanatory variables**	**Distance to facility, media exposure, NHIS subscription, low community economic status, and low community literacy**
Bandwidth	134872.038
Residual Squares	3.618
Effective Number	44.647
Sigma	0.158
AICc	-128.805
R^2	0.623
R^2 Adjusted	0.509

Source: GDHS, 2014

With regards to the distance to a health facility (Figure 4), the north-eastern part of Ghana (shaded red) had a high positive relationship with unskilled birth attendance. This implies that as the proportion of women who identified distance to a health facility as a problem increased, the occurrence of unskilled birth attendance in the north-eastern part of Ghana increases about 44%-77%.

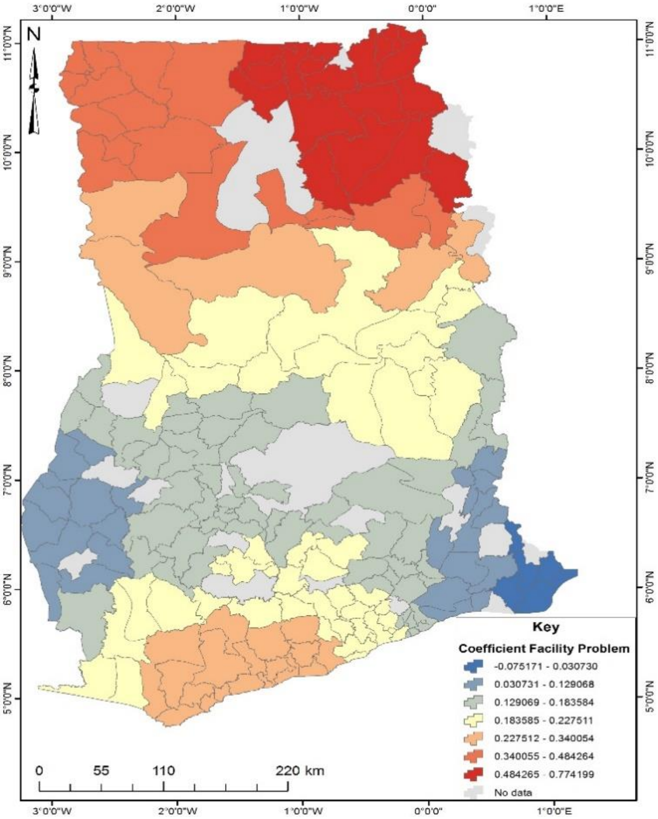

Figure 4: Distance to facility GWR coefficient for predicting unskilled birth attendance in Ghana

Source: GDHS, 2014.

Also, media exposure was a strong predictor of unskilled birth from the north-east through to the mild belt of Ghana (Figure 5). A unit decrease in media exposure would result in about a 30 to 65% increase in unskilled birth from the north-east through to the mild belt of Ghana.

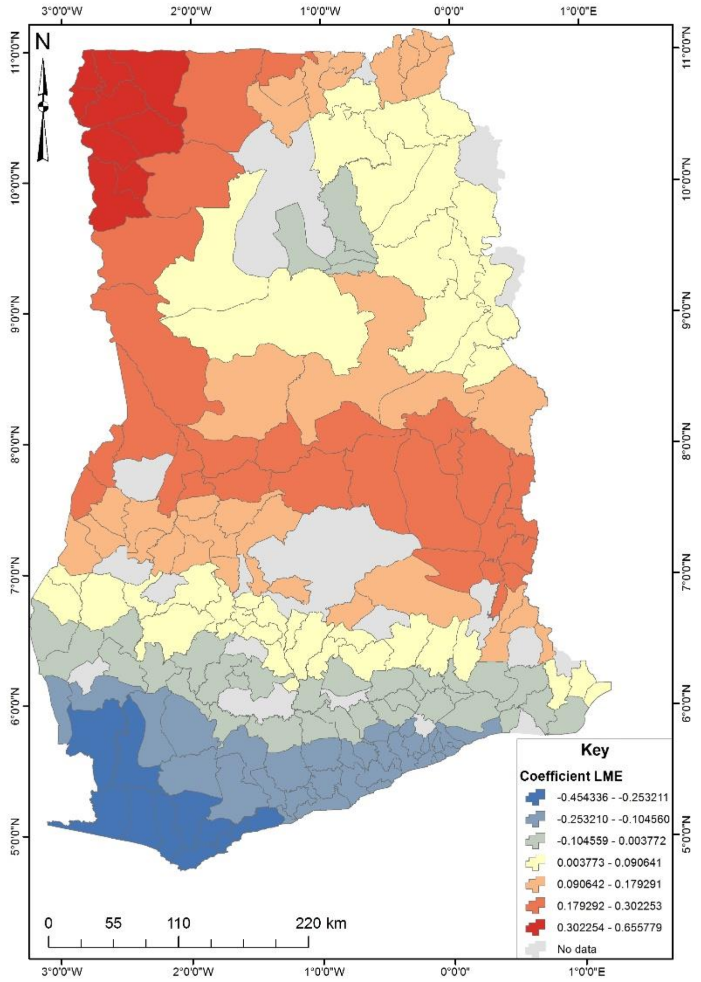

Figure 5: Media exposure GWR coefficient for predicting unskilled birth attendance in Ghana

Source: DHS, 2014

National health insurance subscription was found to be a predictor from west of the northern region through to the southeast of Ghana. A unit increase in national health insurance subscriptions will result in about a 49 to 72% increase in skilled birth attendance in Ghana.

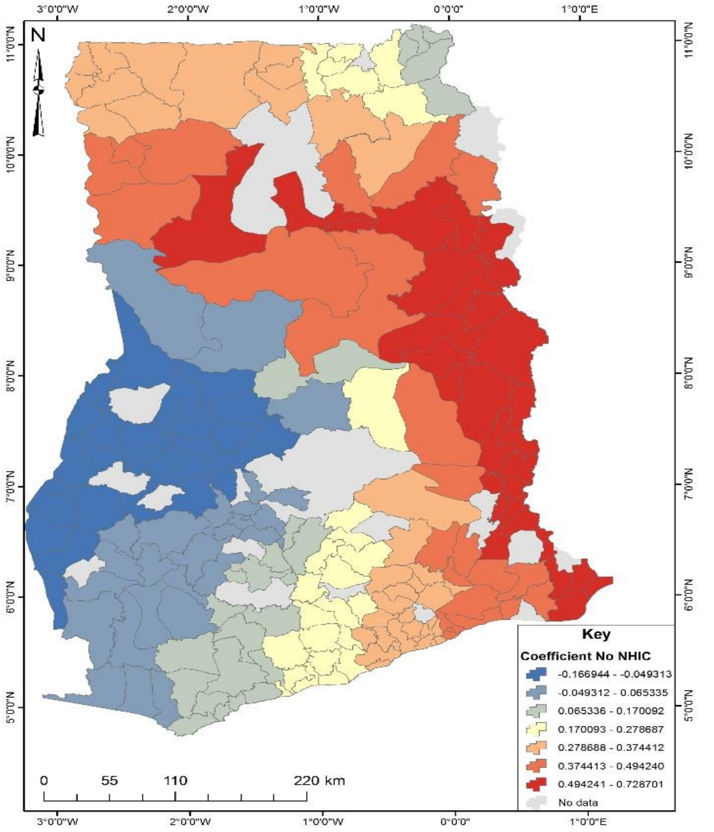

Figure 6: NHIS subscription GWR coefficient for predicting unskilled birth attendance in Ghana.

Source: Computed from GDHS, 2014

Finally, it was found that community literacy level serves as a high predictor of unskilled birth attendance in the north-eastern and southwestern parts of Ghana. As community literacy reduces, unskilled birth attendance increases by about 17 to 25%.

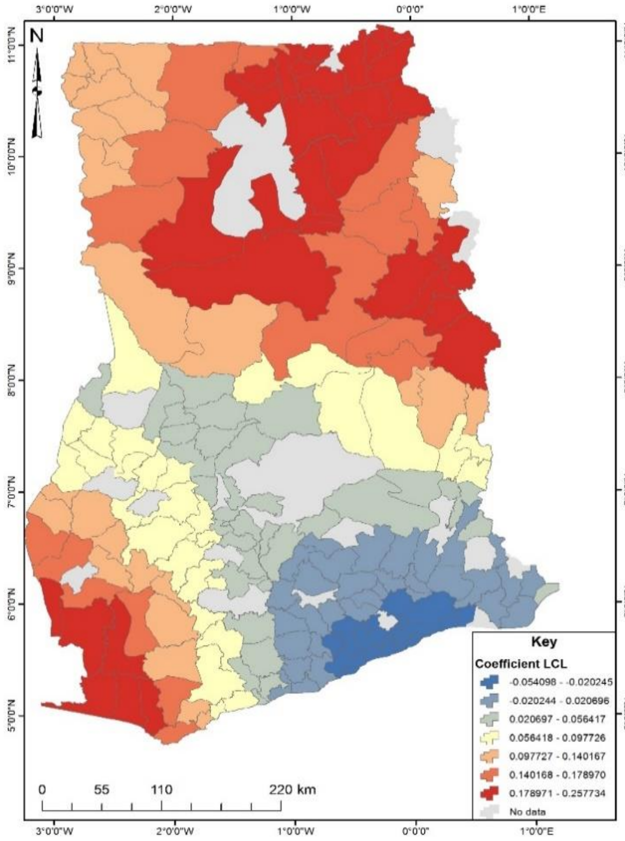

Figure 7: Community Literacy Level GWR coefficient for predicting unskilled birth in Ghana.

Source: GDHS, 2014

Discussion

Among the variables which adjusted for in the ordinary least squares (OLS), the study found that distance to health facility, national health insurance subscription, community economic status, and community literacy as strong predictors of unskilled birth attendance in Ghana for the spatial analysis (i.e. OLSM) while maternal educational level and age, parity, national health insurance subscription, household wealth, media exposure, place of residence, community-level literacy and region of residence were key findings associated with unskilled birth attendance in the non-spatial model. The nonspatial model explained the variation and clustering that exist in unskilled birth attendance in Ghana. For this reason, appreciating the spatial pattern of the analysis of unskilled birth attendance was deemed essential since it helps visualise the phenomenon under study. Therefore, to complement our analysis, we fitted a spatial model to further identify the established pattern and to enable us to provide some justification for the variation in unskilled birth attendance across the country. Generally, the findings (the predictor variables found in the GWR analysis) were like the multilevel analysis conducted in this study. However, our discussions will be based on the results of the spatial model (GWRM) because that is the focus of this study**.**

The study's findings suggested Unskilled birth attendance has a significant spatial variation in Ghana. The hot spot analysis found a total of 16 statistically significant districts at various levels of significance with a high prevalence of unskilled birth attendance. Significant hotspots of unskilled birth attendance were seen in the north-eastern part of Ghana. This finding is in line with findings from Dickson and Amu (17), who reported that women in the Northern and Upper West regions of Ghana were less likely to access skilled assistance during delivery. Variations in skilled birth attendance have been reported in most developing countries as well (33; 31). These variations could be due to the physical inaccessibility and several socio-economic hardships (9), coupled with inequalities in the distribution of scarce resources (45) including inadequate skilled health professionals in remote/border areas of Ghana. In addition, socio-cultural beliefs and practices among women in different districts are potential drivers for the prevalence of unskilled birth participation in Ghana (44; 33). Furthermore, mothers from border districts may have limited access to information concerning maternal health services and other services such as access to school/education that could affect their chances of accessing the services of the skilled birth attendant as was also reported by Antwi and his colleagues (8).

The cluster and outlier analysis was used to verify and complement the hot spot analysis as it allows us to detect both groupings and areas where anomalies exist (26). The cluster and outlier analysis results show aspects that may have been overlooked in the hot spot analysis but are interesting highlights, especially in those areas where diverse types of groupings coexist to influence the outcome of the study. Districts labeled high-low outliers are anomalous districts with a high hotspot for unskilled birth attendance, but they are surrounded by districts with high skilled assisted deliveries. The reason for this outcome is rooted in the uneven distribution of scarce resources. Most areas with fewer populations do not get the needed infrastructural facilities and personnel, since some professionals refuse posting to rural communities in Ghana (2). A similar explanation can be extended to low-high outlier areas where districts with low unskilled birth attendance are surrounded by districts with high unskilled birth attendance.

The GWR analysis revealed a strong positive relationship between mothers who perceived distance to the health facility as a big problem, those not covered by health insurance, those who resided in communities with low literacy and socio-economic levels with unskilled birth attendance in different districts in Ghana. However, exposure to social media could not predict unskilled birth attendance among various districts in the country. For those women who were not covered by health insurance in the hotspot districts, there was a higher likelihood of not visiting the health care facility. This finding is in line with studies conducted in Ghana (7), and elsewhere including Ethiopia (24), and Kenya (38). A possible explanation could be as a result of additional costs incurred if payment for healthcare services is done out of pocket. Similarly, women who are not covered by health insurance in hotspot areas are highly likely to seek alternative health care services different from their peers from cold spot areas. This is supported by our study - areas with higher proportions of people with health insurance subscriptions tend to be cold spots for unskilled births.

The study further found that district-level characteristics are key influencers of unskilled birth delivery in Ghana. A corroboratory to NHIS coverage was the finding that women residing in

districts with low socio-economic levels are more likely to access the services of unqualified and unskilled birth attendants compared to women residing in districts with medium or higher socio-economic levels. This finding is supported by a study done in Nigeria where the utilisation of unskilled birth attendance services was high among women living in rural communities with low socioeconomic status (31) and also in Ethiopia where women from the poorer and poorest household used less of skilled assistance during delivery (33). Generally, women in districts with higher poverty levels are less likely to pay for health insurance subscriptions (34), and the unavailability of health care facilities to readily cater to the health needs of women in poorer districts significantly influence their utilisation of SBA services.

Moreover, participants who perceived distance from the health facility in hotspot districts as a major problem were less likely to seek services for skilled attendant delivery. This is in line with studies done in Ethiopia (33) and rural Nigeria (31). This is because the distance to and from health facilities and lack of transport are crucial factors preventing mothers from seeking and using skilled maternity services.

Conclusion

In Ghana, unskilled birth attendance had spatial variations and clustering across different parts of the country. About 13 randomly distributed districts have high unskilled birth attendance. Districts with high proportions of unskilled birth attendance were detected in the North-Eastern parts of the country. In the GWR analysis, districts with higher proportions of low socio-economic class, low literacy status, highly perceived distance to and from the health facility, and not covered by health insurance were found to have an increased probability of influencing unskilled birth attendance. Due to such spatial variations and clustering, interventions aimed at promoting skilled birth attendance should include primary health care accessibility particularly at the north-eastern part of Ghana, increase in media coverage particularly at the north-eastern part through to the middle belt of Ghana, increase in national health insurance subscription particularly at the northern through to the south eastern part of Ghana, and improvement in community literacy level particularly at the north eastern and southwestern parts of Ghana.

Abbreviations

AICc: Akaike's Information Criterion; AOR: Adjusted Odds Ratio; EAs: Enumeration Areas; GDHS: Ethiopian Demographic and Health Survey OLS (M): Ordinary Least Square (Model); SBA: Skilled Birth Attendant; WHO: World Health Organization; GWR: Geographically Weighted Regression

Declarations

Ethics approval and consent to participate.

This study is a secondary analysis of the de-identified 2014 Ghana Demographic and Health Survey (GDHS), a publicly available dataset. Therefore, ethics approval or consent to participate is not applicable.

Consent for publication

Not applicable

Availability of data and materials

All data relevant to the study are available within the manuscript and anyone who wants the data set can get through requesting online using the link https://www.dhsprogram.com/data/available-datasets.cfm.

Competing interests

The authors declare that they have no competing interests.

Funding

Not applicable

Authors' contributions

KSD and VBB conceived the study. KSD did the statistical analysis and interpreted the results. ENKB and VBB did the spatial analysis. VBB performed the discussion. KSD, VBB, ENKE and BAO, designed the first draft of the manuscript. KSD, VBB, ENKE and BAO, revised the manuscript for intellectual content and gave consent for the version to be published. All authors read and approved the final manuscript to be submitted to this journal.

Acknowledgements

We acknowledge Measure DHS for providing us with the data.

Authors' information

Vincent Bio Bediako[1*], Ebenezer N. K. Boateng[2], Bernard Afriyie Owusu[1], Kwamena Sekyi Dickson[1]

[1]Department of Population and Health, University of Cape Coast, Ghana
[2]Department of Geography and Regional Planning, University of Cape Coast

References

1. Abor PA, Abekah- Nkrumah G, Sakyi K, Adjasi CK, Abor J. The socio- economic determinants of maternal health care utilization in Ghana. International Journal of Social Economics. 2011 Jun 7.
2. Abdulai T, Abobi-Kanbigs DA, Sonlaar KKJ, Adiiboka AJG, Chemogo S. Bridging the Inequitable Distribution of Physicians in Ghana: Factors Medical Students and House Officers at UDS and TTH Will Consider in Accepting Postings to Northern Ghana. Journal of Healthcare Communications [Internet]. 2017 [cited 2021 Mar 4];02(02). Available from: https://healthcare-communications.imedpub.com/bridging-the-inequitable-distribution-of-physicians-in-ghana-factors-medical-students-and-house-officers-at-uds-and-tth-will-consi.php?aid=18892
3. Adatara P, Afaya A, Baku EA, Salia SM, Asempah A. Perspective of Traditional Birth Attendants on Their Experiences and Roles in Maternal Health Care in Rural Areas of Northern Ghana. International Journal of Reproductive Medicine [Internet]. 2018 Oct 1 [cited 2021 Mar 4];2018:1–10. Available from: https://www.hindawi.com/journals/ijrmed/2018/2165627/
4. Addai I. Determinants of use of maternal–child health services in rural Ghana. Journal of biosocial science. 2000 Jan;32(1):1-5.

5. Ameyaw EK, Tanle A, Kissah-Korsah K, Amo-Adjei J. Women's health decision-making autonomy and skilled birth attendance in Ghana. International journal of reproductive medicine. 2016 Dec 26;2016.
6. Ameyaw EK, Dickson KS. Skilled birth attendance in Sierra Leone, Niger, and Mali: analysis of demographic and health surveys. BMC public health. 2020 Dec 1;20(1):164.
7. Amoakoh-Coleman M, Ansah EK, Agyepong IA, Grobbee DE, Kayode GA, Klipstein-Grobusch K. Predictors of skilled attendance at delivery among antenatal clinic attendants in Ghana: a cross-sectional study of population data. BMJ open. 2015 May 1;5(5).
8. Antwi E, Klipstein- Grobusch K, Quansah Asare G, Koram KA, Grobbee D, Agyepong IA. Measuring regional and district variations in the incidence of pregnancy-induced hypertension in Ghana: challenges, opportunities and implications for maternal and newborn health policy and programmes. Tropical medicine & international health. 2016 Jan;21(1):93-100.
9. Apanga PA, Awoonor-Williams JK. Improving Skilled Birth Attendance in Ghana: An Evidence-Based Policy Brief. Journal of Health Care for the Poor and Underserved. 2017;28(3):1056-65.
10. Asamoah BO, Agardh A. Inequality trends in maternal health services for young Ghanaian women with childbirth history between 2003 and 2014. BMJ Open [Internet]. 2017 Feb [cited 2021 Mar 4];7(2):e011663. Available from: https://bmjopen.bmj.com/content/7/2/e011663
11. Austin PC, Merlo J. Intermediate and advanced topics in multilevel logistic regression analysis. Statistics in medicine. 2017 Sep 10;36(20):3257-77.
12. Blanchet NJ, Fink G, Osei-Akoto I. The effect of Ghana's National Health Insurance Scheme on health care utilisation. Ghana medical journal. 2012;46(2):76-84.
13. Bongaarts J. WHO, UNICEF, UNFPA, World Bank Group, and United Nations Population Division Trends in Maternal Mortality: 1990 to 2015 Geneva: World Health Organization, 2015
14. Budu E. Predictors of home births among rural women in Ghana: analysis of data from the 2014 Ghana Demographic and Health Survey. BMC Pregnancy and Childbirth. 2020 Dec;20(1):1-8.
15. Trends in maternal mortality: 1990 to 2015. World Health Organization [Internet]. 2020 Jan 24 [cited 2021 Mar 4]; Available from: https://www.who.int/reproductivehealth/publications/monitoring/maternal-mortality-2015/en/
16. de Bernis L, Sherratt DR, AbouZahr C, Van Lerberghe W. Skilled attendants for pregnancy, childbirth and postnatal care. British medical bulletin. 2003 Dec 1;67(1):39-57.
17. Dickson KS, Amu H. Determinants of skilled birth attendance in the northern parts of Ghana. Advances in Public Health. 2017 Jan 1;2017.
18. Dixon J, Tenkorang EY, Luginaah I. Ghana's National Health Insurance Scheme: helping the poor or leaving them behind?. Environment and Planning C: Government and policy. 2011 Dec;29(6):1102-15.
19. Dotse- Gborgbortsi W, Tatem AJ, Alegana V, Utazi CE, Ruktanonchai CW, Wright J.
Spatial inequalities in skilled attendance at birth in Ghana: a multilevel analysis integrating health facility databases with household survey data. Tropical Medicine & International Health [Internet]. 2020 Jul 18 [cited 2021

Mar 4];25(9):1044–54. Available from: https://onlinelibrary.wiley.com/doi/full/10.1111/tmi.13460?af=R
20. Jehu-Appiah C, Aryeetey G, Spaan E, De Hoop T, Agyepong I, Baltussen R. Equity aspects of the National Health Insurance Scheme in Ghana: Who is enrolling, who is not and why?. Social science & medicine. 2011 Jan 1;72(2):157-65.
21. Khan SM, Singh K. The Association between health insurance coverage and skilled birth attendance in Ghana: a national study. Maternal and child health journal. 2016 Mar 1 ;20(3):534-41.
22. Maduka O, Ogu R. Preventing Maternal Mortality during Childbirth: The Scourge of Delivery with Unskilled Birth Attendants. Childbirth. 2020 Mar 18:119.
23. Merlo J, Wagner P, Ghith N, Leckie G. An original stepwise multilevel logistic regression analysis of discriminatory accuracy: the case of neighbourhoods and health. PloS one. 2016 Apr 27;11(4):e0153778.
24. Mezmur M, Navaneetham K, Letamo G, Bariagaber H. Individual, household and contextual factors associated with skilled delivery care in Ethiopia: evidence from Ethiopian demographic and health surveys. PLoS One. 2017 Sep 14;12(9):e0184688.
25. Patel AB, Simmons EM, Rao SR, Moore J, Nolen TL, Goldenberg RL, Goudar SS, Somannavar MS, Esamai F, Nyongesa P, Garces AL. Evaluating the effect of care around labor and delivery practices on early neonatal mortality in the Global Network's Maternal and Newborn Health Registry. Reproductive health. 2020 Nov;17(2):1-1.
26. Sánchez-Martín JM, Rengifo-Gallego JI, Blas-Morato R. Hot spot analysis versus cluster and outlier analysis: an enquiry into the grouping of rural accommodation in Extremadura (Spain). ISPRS International Journal of Geo-Information. 2019 Apr;8(4):176.
27. Sarpong N, Loag W, Fobil J, Meyer CG, Adu- Sarkodie Y, May J, Schwarz NG. National health insurance coverage and socio- economic status in a rural district of Ghana. Tropical medicine & international health. 2010 Feb;15(2):191-7.
28. Say L, Chou D, Gemmill A, Tunçalp Ö, Moller AB, Daniels J, Gülmezoglu AM, Temmerman M, Alkema L. Global causes of maternal death: a WHO systematic analysis. The Lancet global health. 2014 Jun 1;2(6):e323-33.
29. Shifti DM, Chojenta C, Holliday EG, Loxton D. Application of geographically weighted regression analysis to assess predictors of short birth interval hot spots in Ethiopia. PloS one. 2020 May 29;15(5):e0233790.
30. Singh K, Osei-Akoto I, Otchere F, Sodzi-Tettey S, Barrington C, Huang C, Fordham C, Speizer I. Ghana's National Health insurance scheme and maternal and child health: a mixed methods study. BMC health services research. 2015 Dec;15(1):1-3.
31. Solanke BL, Rahman SA. Multilevel analysis of factors associated with assistance during delivery in rural Nigeria: implications for reducing rural-urban inequity in skilled care at delivery. BMC pregnancy and childbirth. 2018 Dec;18(1):1-5.
32. Tampah-Naah AM, Osman A, Kumi-Kyereme A. Geospatial analysis of childhood morbidity in Ghana. Plos one. 2019 Aug 30;14(8):e0221324.
33. Teshale AB, Alem AZ, Yeshaw Y, Kebede SA, Liyew AM, Tesema GA, Agegnehu CD. Exploring spatial variations and factors associated with skilled birth attendant delivery in Ethiopia: geographically weighted regression and multilevel analysis. BMC public health. 2020 Dec;20(1):1-9.

34. Umeh CA, Feeley FG. Inequitable access to health care by the poor in community-based health insurance programs: a review of studies from low-and middle-income countries. Global Health: science and practice. 2017 Jun 27;5(2):299-314.
35. unstats | Millennium Indicators [Internet]. Un.org. 2015 [cited 2021 Mar 4]. Available from: http://mdgs.un.org/unsd/mdg/SeriesDetail.aspx?srid=570&crid=288
36. UNICEF. (2018). Maternal and Newborn Health Disparities: Key Facts. Available at https://www.ghanahealthservice.org/downloads/NEWBORN0001.pdf accessed, 22nd January 2021.
37. Levels and Trends in Child Mortality Report 2015 | United Nations Population Division | Department of Economic and Social Affairs [Internet]. Un.org. 2015 [cited 2021 Mar 4]. Available from: https://www.un.org/en/development/desa/population/publications/mortality/child-mortality-report-2015.asp
38. Were LP, Were E, Wamai R, Hogan J, Galarraga O. The Association of Health Insurance with institutional delivery and access to skilled birth attendants: evidence from the Kenya Demographic and health survey 2008–09. BMC health services research. 2017 Dec;17(1):1-0.
39. Witter S, Garshong B. Something old or something new? Social health insurance in Ghana. BMC International health and human rights. 2009 Dec;9(1):1-3.
40. World Health Organization. Making pregnancy safer: the critical role of the skilled attendant: a joint statement by WHO, ICM and FIGO. World health organization; 2004.
41. World Health Organization: WHO. Maternal mortality [Internet]. Who.int. World Health Organization: WHO; 2019 [cited 2021 Mar 4]. Available from: https://www.who.int/news-room/fact-sheets/detail/maternal-mortality
42. World Health Organization. Regional Office for the Western Pacific. Sustainable development goals (SDGs) : Goal 3. Target 3.1 : By 2030, reduce the global maternal mortality ratio to less than 70 per 100,000 live births [poster]. Manila : WHO 2016 Regional Office for the Western Pacific. https://apps.who.int/iris/handle/10665/208272
43. Yakoob MY, Ali MA, Ali MU, Imdad A, Lawn JE, Van Den Broek N, Bhutta ZA. The effect of providing skilled birth attendance and emergency obstetric care in preventing stillbirths. BMC public health. 2011 Dec;11(3):1-8.
44. Yarney L. Does knowledge on socio-cultural factors associated with maternal mortality affect maternal health decisions? A cross-sectional study of the Greater Accra region of Ghana. BMC Pregnancy and Childbirth [Internet]. 2019 Jan 28 [cited 2021 Mar 4];19(1). Available from: https://bmcpregnancychildbirth.biomedcentral.com/articles/10.1186/s12884-019-2197-7
45. Zere E, Kirigia JM, Duale S, Akazili J. Inequities in maternal and child health outcomes and interventions in Ghana. BMC public health. 2012 Dec;12(1):1-0.
