# Supplementary material for: Multilevel geospatial analysis of factors associated with unskilled birth attendance in Ghana
Source: PLoS One. 2021 Jun 25;16(6):e0253603. doi: 10.1371/journal.pone.0253603 (PMC8232528; doi:10.1371/journal.pone.0253603)
Supplement: Attachment — Submitted filename: Response to Reviewers.doc [file pone.0253603.s006.doc]

**Reviewer’s Comments**

**Multilevel geospatial analysis of unskilled birth attendant in Ghana**

The authors examined an important aspect maternal health especially in Ghana. However, I have some few comments for consideration.

**General observations**

The topic should be revised to “Multilevel geospatial analysis of factors associated with unskilled birth deliveries in Ghana.”

The manuscript requires proofreading to improve upon the English Language.

Referencing has been done haphazardly. There is no coherence right from the start of writing. E.g. in the Background, the first reference was numbered (41), then followed with (36) and (13). This inconsistency is obvious throughout the whole work.

**Background**

Paragraphs 2 and part of paragraph 3 should not be part of the background. These paragraphs highlights issues on the definition of skilled births and some related statistics of skilled births. The focus of the study is unskilled births. Therefore, the background should succinctly concentrate on unskilled births. This will distinguish it from studies that have examined skilled births in Ghana.

Also, the opening sentence of the last paragraph of the background suggests a special interest in a particular ecological zone in Ghana. This I believe is the case. The study considered the country. This too needs to be revised. Rather highlight unskilled birth situations in the country.

The whole background should be revised in a coherent manner to inform the reader exactly what the study is meant to examine, thus unskilled births in Ghana.

**Methods**

**Source of data**

“For the study, women with birth history who had given birth up to five years before the survey were included”. These inclusion criteria is not clear. With a period of five years, a woman can comfortably give birth to at least two children assuming there are no any medical complications. And these births can occur at home or at health facility or both at home or both at a health facility. So, which one of these children should the study be on? For uniform selection of women, only the last birth of the women aged 15-49 years preceding the survey should be included in the study. If this done, it will certainly affect the current sample size of 4,290 women; meaning re-analysis is required.

**Response**

The target population was **women** who gave birth 5 years before the survey and not children. Only the last birth of the women aged 15-49 years preceding the survey should be included in the study. This section has been reviewed to make it clearer.

**Outcome variable**

How was the outcome variable coded?

**Response**

A Table has been developed to show how the variables were coded and measured.

**Explanatory variable**

It will be interesting for the authors to indicate the various responses of the variables as captured in the GDHS and then indicate how each of them was recoded, where necessary.

**Response**

A Table has been developed to show how the variables were coded and measured.

**Statistical analysis**

Authors should indicate how variables were selected for the regression models. It seems preference was given to some variables at each stage of the modelling.

**Response**

Based on literature, the individual variables were introduced first, and then the community variables were co. the last model is the full model that combined the individual and community variables.  Model 1 was an empty table, where model 2 looked at the relationship between the individual variable and the outcome variable. Model 3 looked at the relationship between community variables and the outcome variable. Model 4 was the complete model that looked at the relationship with both the individual and community variables and the outcome variable.

**Spatial analysis**

This section is too detailed. It should be summarized in two paragraphs for purposes of sustaining the interest of reader and also making the content simple.

**Response**

This section has been reduced into two paragraphs as suggested.

**Results**

**Background characteristics**

- To refer to a table in text, state it as (Table 1) and not as (see Table 1).

Response

Revision has been done.

- On Table 1, proportion for ‘Richest’ category of wealth status is missing.

Response

The proportion for ‘Richest’ category of wealth status has been added

**Spatial distribution on unskilled births attendants**

Some of the spatial figures and tables can be on supplementary sheets. Some of these include:

a. Fig. 1: Spatial autocorrelation of unskilled births in Ghana
b. Table 4: OLS Diagnostics
c. Table 5: GWR model for unskilled birth attendance in Ghana

**Response**

The reference (see Table) has been corrected to (Table). The proportion for the richest has been filled. The suggestion has been implemented where Figure 1, Table 4 and Table 5 have been moved to appendix.

The authors failed to mention the specific districts that are associated with unskilled births in Ghana. It is important to mention these districts to better inform policy makers and readers where unskilled births are high within the country.

**Response**

All the districts that were identified as hotspot for unskilled birth attendance has been mentioned. Also, the districts involved in cold spot and cluster and outlier analysis has been reported.

The discussion was well presented however the referencing style is not appropriate.

Conclusion too was well presented.

**Response**

The referencing style has been corrected to the standard required by the journal.
